# Characteristics of Twitter Use by State Medicaid Programs in the United States: Machine Learning Approach

**DOI:** 10.2196/18401

**Published:** 2020-08-17

**Authors:** Jane M Zhu, Abeed Sarker, Sarah Gollust, Raina Merchant, David Grande

**Affiliations:** 1 Division of General Internal Medicine Oregon Health and Science University Portland, OR United States; 2 Department of Biomedical Informatics Emory University Atlanta, GA United States; 3 Division of Health Policy and Management University of Minnesota Minneapolis, MN United States; 4 Center for Digital Health University of Pennsylvania Philadelphia, PA United States; 5 Division of General Internal Medicine University of Pennsylvania Philadelphia, PA United States

**Keywords:** medicaid, public health, health communication, community engagement, social media

## Abstract

**Background:**

Twitter is a potentially valuable tool for public health officials and state Medicaid programs in the United States, which provide public health insurance to 72 million Americans.

**Objective:**

We aim to characterize how Medicaid agencies and managed care organization (MCO) health plans are using Twitter to communicate with the public.

**Methods:**

Using Twitter’s public application programming interface, we collected 158,714 public posts (“tweets”) from active Twitter profiles of state Medicaid agencies and MCOs, spanning March 2014 through June 2019. Manual content analyses identified 5 broad categories of content, and these coded tweets were used to train supervised machine learning algorithms to classify all collected posts.

**Results:**

We identified 15 state Medicaid agencies and 81 Medicaid MCOs on Twitter. The mean number of followers was 1784, the mean number of those followed was 542, and the mean number of posts was 2476. Approximately 39% of tweets came from just 10 accounts. Of all posts, 39.8% (63,168/158,714) were classified as general public health education and outreach; 23.5% (n=37,298) were about specific Medicaid policies, programs, services, or events; 18.4% (n=29,203) were organizational promotion of staff and activities; and 11.6% (n=18,411) contained general news and news links. Only 4.5% (n=7142) of posts were responses to specific questions, concerns, or complaints from the public.

**Conclusions:**

Twitter has the potential to enhance community building, beneficiary engagement, and public health outreach, but appears to be underutilized by the Medicaid program.

## Introduction

Approximately 20% of online adults use Twitter [1], a social media platform that allows users to share short messages (“tweets”). With over 500 million tweets per day on topics including health-related issues, Twitter is a potentially valuable tool for public health officials to engage the public. Prior studies suggest that overall social media use is independent of educational attainment, race/ethnicity, education, income, and health care access [2]. Twitter use is also significantly higher in young adults compared with older age groups, which maps well to the Medicaid population, of which 93% are aged <65 years and 80% are aged <45 years [3]. However, evidence suggests that these platforms are currently underutilized. A minority of local public health departments and federal health agencies in the United States use Twitter [4], and these accounts typically have low public engagement [5].

Twitter may be particularly useful for state Medicaid programs, which together constitute the single largest source of public health insurance in the United States, covering 72 million children, older adults, people with disabilities, and low-income populations. Medicaid is currently undergoing a variety of program and policy changes, including eligibility expansion to low-income childless adults; establishment of work requirements for some enrollees; delivery system reforms like accountable care organizations and value-based payment models; and addressing emerging public health priorities like substance use disorder treatment. Given this context, there are significant opportunities for state agencies to better understand, respond to, and engage with the diverse needs of Medicaid-eligible and Medicaid-enrolled populations [6] and to communicate with the general public as a key stakeholder. Despite this potential, to our knowledge no prior study has examined the extent to which Medicaid agencies and health plans are using Twitter to communicate with the public.

## Methods

We identified active Twitter profiles of state Medicaid agencies and Medicaid managed care organizations (MCOs), which are health insurance plans that contract with states to provide Medicaid health benefits and related services. We included the latter group because more than 70% of Medicaid beneficiaries receive their care through comprehensive managed care plans. To identify Medicaid MCOs, we used the Kaiser Family Foundation’s publicly available Medicaid Managed Care Market Tracker [7], which lists all Medicaid MCOs and their parent firms across all 50 states and the District of Columbia. For each state, we searched Twitter, agency websites, and Google to identify and verify relevant Medicaid agency and MCO accounts. Our focus was on state-level Medicaid programs and plans, so we excluded accounts of higher-level governmental agencies (eg, Department of Health and Human Services), parent firms of managed care plans operating across multiple states, and professional accounts of individual Medicaid administrators and leadership.

We used the Twitter public application programming interface [8] to collect a total of 158,714 public posts by the identified accounts, spanning March 2014 through June 2019. To conduct content analysis on these tweets, we manually coded 800 tweets and developed a coding scheme with 5 broad categories of tweet content (Table 1). Three coders independently coded identical samples of tweets, and resolved disagreements and updated the coding guidelines via discussion. Two coders then reviewed a new set of 5338 tweets (excluding retweets), yielding an intercoder agreement of 0.78 using Cohen over 998 overlapping tweets. Tweets that could not be categorized as belonging to one of the 5 categories were labeled “Other” (n=198).

We used the coded tweets to train and evaluate supervised machine learning algorithms, experimenting with four approaches: naive Bayes, support vector machines, random forests, and an ensemble of these three classifiers, which combined the predictions of the independently trained classifiers using predetermined rules [9]. The ensemble classifier obtained the best performance when evaluated using the manually annotated labels, with an accuracy of 74.1%, and therefore we used this classifier to automatically classify all the posts in our data set.

## Results

We identified 96 Twitter accounts, including 15 state Medicaid agencies and 81 Medicaid MCOs (out of 51 total state Medicaid agencies and 323 Medicaid MCOs). Among these accounts, the mean number of followers was 1784 (range 2-38,352, median 1434), the mean number of those followed was 542, and the mean number of posts was 2476. Twitter accounts had been active for a mean of 79 months, and approximately 39% of tweets came from just 10 accounts.

The most active accounts in terms of number of tweets were those of health plans, including Fidelis (New York, @FidelisCare, 14,748), HAP Midwest Health Plan Inc (Michigan, @hapmichigan, 12,598), Hawaii Medical Service Association (Hawaii, @AskHMSA, 12,568), Blue Cross Blue Shield of Texas (Texas, @BCBSTX, 12,157), and UPMC Health Plan (Pennsylvania, @UPMCHealthPlan, 8302). Top accounts in terms of follower count were also health plans, including Fidelis (38,352), UPMCHealthPlan (14,845), Blue Cross Blue Shield of Texas (9355), Seton Health Plan (Texas, @setonfamily, 8326), and Anthem Blue Cross Partnership Plan (California, @AnthemBC_News, 7173). In comparison, top Medicaid agencies in terms of follower count included Ohio (@OhioMedicaid, 3165), Massachusetts (@MassHealth, 2223), Washington (@WA_Health_Care, 1858), Colorado (@CHCPF, 1794), and Georgia (@GADCH, 1596).

Of all posts (N=158,714), 39.8% (n=63,168) were classified as general public health education and outreach; 23.5% (n=37,298) constituted outreach about specific Medicaid policies, programs, services, or events; 18.4% (n=29,203) contained relationship-building content including organizational promotion of staff and activities; and 11.6% (n=18,411) contained general news and news links (Table 1). Only 4.5% (n=7142) of the tweets were customer service responses to specific questions, concerns, or complaints from other users. Additionally, 2.2% (n=3492) of the tweets did not fit within these categories and were classified as “Other.”

The majority of posts received little or no engagement from the public, with only 1% of tweets having 9 or more likes, or 6 or more retweets. Tweets with more active engagement were more likely to contain words that conveyed actions directed at the public, like “email,” “assist,” “send,” and “contact” (see sample tweets in Table 1).

**Table 1:** Frequency and types of posts from Medicaid agencies and managed care organizations on Twitter (N=158,714)^a^.

Category	Definition	Example	Posts, n (%)
Public health education	Public health announcements about prevention, disease conditions, vaccinations, resources, and provision of general health-related information or health-seeking behaviors.	Learn the symptoms for each type of gynecologic cancer. If you have some of the symptoms, talk to your provider. https://t.co/UPxaBrxY5R	63,168 (39.8)
Outreach about specific Medicaid policies and programs	Information to raise awareness of specific services, programs, and events offered by Medicaid, directed toward consumers	All SoonerCare members are required to have a valid Oklahoma mailing address on file 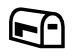 Don't have your Oklahoma address listed on your account? Update your information today at https://t.co/AEkZDU7W4E. Unsure if your insurance is still active or if you need to reapply? Call #HUSKYHealth at 1.800.859.9889 for help.	37,298 (23.5)
Relationship building or promotional	Posts directed at a general audience that intends to elicit consumer engagement (responses, replies, likes, feedback), including promotion of agency staff and accomplishments, and greetings.	What would you want to improve about your Medicaid program? Like us on Facebook. Get access to health tips, recipes, and events happening around the state. Visit: https://t.co/0XI6PEfhcq	29,203 (18.4)
General news and announcements	Press releases, links to news articles, general announcements	New post (Three regional agencies team up to support MassHealth consumers with disabilities, complex medical needs) has been published on Advocate News Online - https://t.co/JMfIU0sdhH	18,411 (11.6)
Customer service and responses to constituents	Responses from the plan or agency directed toward a specific question or complaint raised by a consumer	@[redacted] Renewals are done yearly. You will be notified when it’s time to renew your coverage.	7142 (4.5)

^a^Note that 2.2% (n=3492) of the tweets did not fit within these categories and were classified as “Other,” which is not shown in this table.

## Discussion

### Principal Findings

Social media is one way for Medicaid programs to reach constituents and to launch low-cost health promotion and public engagement campaigns in the face of resource constraints. Our results suggest that despite Twitter’s reach among the general public, it is underutilized in the Medicaid program, compared to other public health organizations [10,11].

Twitter has the potential to enhance community building and engagement, which is increasingly a priority for Medicaid programs. Prior work found that two-way communication on Twitter between public health entities and constituents led to an increase in action and awareness that, in turn, resulted in an improvement in community health [12]. However, many public health entities use social media simply to broadcast information without engaging audiences [4,10]. While we found that some Medicaid programs are using Twitter, the majority have relatively few followers and overall low engagement with the public. A number of studies point to methods to increase public engagement on Twitter. A study of US children’s hospitals using social media found improvements in engagement when posts included pictures and content featuring patient narratives and community partnerships [13]. Other studies in the health care sector have found increased engagement with posts that feature hashtags, URLs, and user mentions [4].

Our study has limitations. Although we used multiple search strategies to verify accounts, we may be missing some active Medicaid accounts. We also excluded a number of umbrella state agencies on Twitter whose activities may include some degree of Medicaid program oversight and outreach, which may lead to underestimation of Medicaid agency presence and activity. Additionally, we used machine learning algorithms to automate content analysis of a large set of tweets, and there may be some misclassification of text across coding categories. Finally, our study focused on Twitter and is not generalizable to other social media platforms that Medicaid agencies and health plans may be using to engage with the public.

### Conclusions

Changes to the Medicaid program have accelerated a number of efforts to increase consumer engagement around disease and care management, cost-sharing, and health care behavior change. As state Medicaid programs confront important public health challenges and expand to serve new populations, social media represents an important and underutilized tool for engaging both Medicaid-eligible individuals and the general public. Future research is needed to understand how social media platforms like Twitter can help these programs improve community engagement around public health programming and interventions.

## Abbreviations

MCO: managed care organization
